# The Mechanism of Ovule Abortion in Self-Pollinated ‘Hanfu’ Apple Fruits and Related Gene Screening

**DOI:** 10.3390/plants13070996

**Published:** 2024-03-30

**Authors:** Haiyang Wei, Baoan Wang, Ya Xu, Wenqi Fan, Manyu Zhang, Fuli Huang, Chenxi Shi, Tianzhong Li, Shengnan Wang, Shengyuan Wang

**Affiliations:** College of Horticulture, China Agricultural University, Beijing 100193, China

**Keywords:** apple, self-pollination, ovule abortion, SMART-seq

## Abstract

Apples exhibit S-RNase-mediated self-incompatibility and typically require cross-pollination in nature. ‘Hanfu’ is a cultivar that produces abundant fruit after self-pollination, although it also shows a high rate of seed abortion afterwards, which greatly reduces fruit quality. In this study, we investigated the ovule development process and the mechanism of ovule abortion in apples after self-pollination. Using a DIC microscope and biomicroscope, we found that the abortion of apple ovules occurs before embryo formation and results from the failure of sperm–egg fusion. Further, we used laser-assisted microdissection (LAM) cutting and sperm and egg cell sequencing at different periods after pollination to obtain the genes related to ovule abortion. The top 40 differentially expressed genes (DEGs) were further verified, and the results were consistent with switching the mechanism at the 5′ end of the RNA transcript (SMART-seq). Through this study, we can preliminarily clarify the mechanism of ovule abortion in self-pollinated apple fruits and provide a gene reserve for further study and improvement of ‘Hanfu’ apple fruit quality.

## 1. Introduction

Seed plants are the highest group of plant species in the plant kingdom. Seeds develop from ovules after flowering and after the process of pollination and fertilization [1]. Typically, seeds have three constituent parts: the seed coat, the embryo, and the endosperm [2]. *Malus domestica* is a common seed plant that does not normally produce fruit by means of self-pollination; only a few self-pollinating apple varieties have been discovered after rigorous screening processes [3,4,5,6,7,8,9,10,11]. ‘Hanfu’ is a cultivar that produces abundant fruit after self-pollination. However, self-pollinated ‘Hanfu’ fruits show a significant rate of abortion, which causes an imbalanced fruit morphology and adversely affects fruit quality. In seed plants, from the time the pollen falls on the stigma to the completion of fertilization, the following processes also occur: pollen adhesion and hydration, pollen tube growth, sperm discharge, and fusion of male and female gametes [12,13,14,15,16]. Mature pollen grains attach to the stigma by active or passive means and after hydration pollen grains germinate on the stigma [17,18,19]. After being guided primarily by ovule signals, the pollen tube grows along the transmitting tissue at the base of the style and enters the ovule via the micropyle [20,21,22,23,24]. Once it has entered the ovule, the pollen tube ruptures and discharges two sperm cells [25,26]. Upon the recognition of male and female gametes within the embryo sac, a sperm cell proceeds to fuse with the egg cell, resulting in the embryo formation. Another sperm cell fuses with the central cell, leading to the endosperm formation [12]. Problems at any of these steps can lead to the failure of fertilization. For example, the ‘1913’ pear cultivar has issues at the time of fusion [27]. Previous studies have reported that the *EC1* (egg cell-specific expressed genes) is a group of secretory peptides located in the egg cell, with five members, EC1.1–EC1.5, which are located in the spherical vesicle-like structure of the egg cell. Upon sperm arrival, EC1 are exocytosed, providing the impetus for separation of the sperm pair [28,29]; mediated by the spermatocyte-specific expression of the membrane proteins DOMAIN OF UNKNOWN FUNCTION 679 (DUF679) membrane proteins (DMP8 and DMP9) [30,31,32], HAPLESS 2 (HAP2), also known as GENERATIVE CELL-SPECIFIC 1 (GCS1) which move to the sperm surface [33,34,35]. Cell fusion begins when the adhesion protein GEX2 on the sperm membrane binds to the unknown GEX2-binding protein on the egg cell [36,37,38]. Programmed cell death in endosperm and seed coat has been found in grapes [39]. However, the fundamental reason for ovule abortion in apple remains unclear.

In our previous study, both self-pollination and cross-pollination of ‘Hanfu’ led to ovule abortion [4]. However, the proportion of ovule abortion in self-pollinated ‘Hanfu’ was significantly higher than in cross-pollinated ‘Hanfu’. In this study, we investigated the phenotypic and microscopic differences between alive ovules and abortive ovules in the pollination combinations of ‘Hanfu’ × ‘Hanfu’ and ‘Hanfu’ × ‘RallsJanet’. By paraffin sectioning and microscopic observation, it was found that the 8 days after pollination (DAP) was the best period to study ovule abortion in ‘Hanfu’. Laser-assisted microdissection (LAM) was performed to sample sperm and egg cell during ovule abortion for further SMART-seq. The aim of this study was to provide a theoretical basis and gene reserve for future research on the mechanism of ovule abortion in seed plants, as well as a framework for enhancing the quality of apple fruits.

## 2. Materials and Methods

### 2.1. Experimental Materials

The parentage of the ‘Hanfu’ apples used in this study can be traced back to ‘Dongguang’ and ‘Fuji’ cultivars grown at Shangzhuang Experimental Station in the Haidian District of Beijing. The experiment utilized pollen from ‘Hanfu’ and ‘RallsJanet’ cultivars. The anthers collected from the flowers were stored at room temperature for natural anther dehiscence, which took 4–5 days. After dehiscence, the anthers were put into a vial and stored in a color-changing silica gel desiccant, then refrigerated at −20 °C for future use, and was then tested in medium containing 10% (*w*/*v*) sucrose, 0.01% (*w*/*v*) H_3_BO_3_, and 0.015% (*w*/*v*) CaCl_2_.

### 2.2. Pollination Methods

We emasculated and bagged the inflorescences used in all three pollination combinations roughly one week before anthesis. We plucked some of the flowers and removed the anthers into paper containers for natural pollen dispersal at room temperature. To accelerate pollen dispersal, we positioned an electric heater near the sample. ‘Hanfu’ and ’RallsJanet’ pollen was used to artificially pollinate ‘Hanfu’ flowers using rubber tips. Then, two flowers were kept in each inflorescence and covered with a bag. We conducted experiments with three pollination combinations, ’Hanfu’ × ’Hanfu’, ’Hanfu’ × ’RallsJanet’, and ’Hanfu’ (non-pollination).

### 2.3. Seed Transparent Treatment and DIC Observation

Every two days after pollination (DAP), ovaries were sampled, fixed in an FAA fixative, and stored in a refrigerator at 4 °C for backup. The ovaries were dissected using a knife to obtain ovules. These underwent dehydration using 70%, 85%, and 100% ethanol, the last of which was repeated thrice. The process lasted for 20 min for each ethanol concentration. Following this, the ovules were immersed in 50% methyl salicylate with ethanol (equal volume) for two hours. Next, they were placed in 100% methyl salicylate for 12–48 h, pressed, and examined using a DIC microscope.

### 2.4. Seed Dissection and Observation

During every period, fifty ovaries were chosen at random from the FAA fixative, which consisted of formaldehyde, glacial acetic acid, and 70% alcohol in a ratio of 5:5:90, respectively. These ovaries were then dissected under a microscope, and their ovules were removed and organized chronologically. A Fujifilm X-T200 camera (Fujifilm holdings, Tokyo, Japan) was utilized for photographic observation, and image software was implemented for comparative analysis of the maximum cross-sectional area.

### 2.5. Paraffin Sections and LAM

Ovules were dissected under a dissecting microscope from ovaries fixed for 24 h in Carnot fixative (anhydrous ethanol: acetic acid = 3:1) and removed. After passing through osmotic wax, embedding, and sectioning, they were dehydrated through an ethanol series and then embedded in paraffin. The embedded tissue was sectioned to a thickness of 4–8 μm using a rotary slicer. Sections were rehydrated, stained with Alcian blue and nuclear solid red [27], observed using an Olympus CX31 light microscope, and photographed with panoramic scanning at Scientific Compass Company, Beijing, China.

Because the dyes Alcian blue and nuclear solid red interfere with RNA extraction, paraffin sections were serially sectioned and placed on a membrane slide (4 μm PEN film, Life Technologies, New York, NY, USA). Staining and light microscopy of 1 section was performed every 2 sections, and if sperm cells and oocytes could be observed, the same positions in the 2 sections spaced above and below were cut for collection [40,41,42].

### 2.6. RNA Extraction and Transcriptome Analysis

RNA was extracted from the collected cells using the Qiagen RNeasy Micro Kit RNA extraction kit. RNA concentration was determined using an ND-1000 Nano Drop spectrophotometer (Thermo Fisher Scientific, Waltham, MA, USA). RNA was sent to Beirui Biotechnology company(Beijing, China) for SMART-seq, on the PacBio Sequel Ⅱplatform. The genome data used in this paper was obtained from GDR (https://www.rosaceae.org/species/malus_x_domestica_HFTH1/genome_v1.0), accessed on 11 December 2023.

## 3. Results

### 3.1. Abortion of Apple Ovules Due to Failure of Embryo Formation

**Figure 1:**
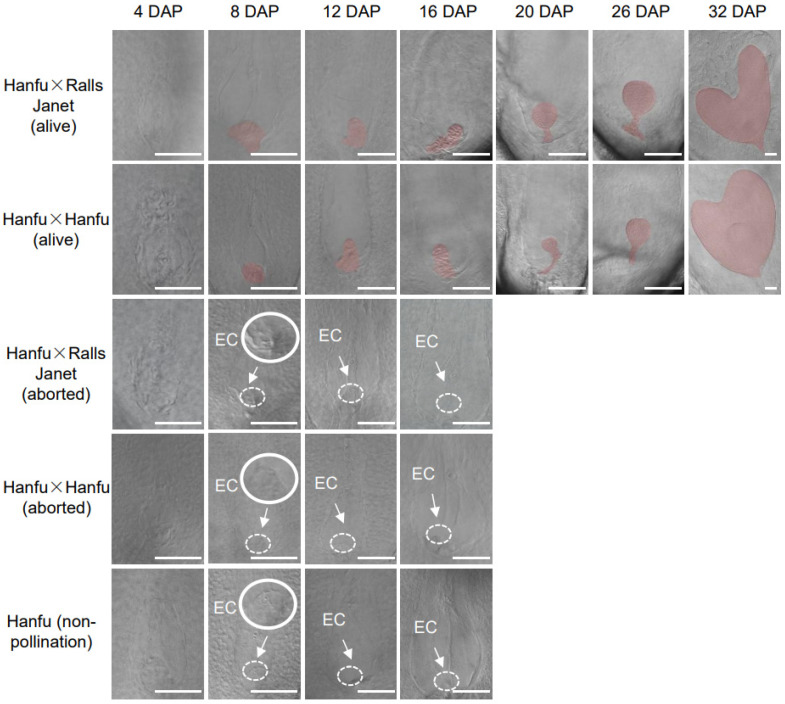
Ovule development process in apple after pollination in ‘Hanfu’ × ‘RallsJanet’ (alive), ‘Hanfu’ × ‘Hanfu’ (alive), ‘Hanfu’ × ‘RallsJanet’ (aborted), ‘Hanfu’ × ‘Hanfu’ (aborted), and ‘Hanfu’ (non-pollination). Red areas represent the embryo. White dotted line area represents the egg cell (EC); solid white line represents the enlarged egg cell. DAP, days after pollination. Scale bars, 100 μm.

To explore sperm–egg cell union and ovule development in self-pollinating apples, the self-compatibility of the apple variety ‘Hanfu’ was used as the research material. Before 4 DAP, it was possible to observe unfertilized egg cells and large central cells in the embryo sacs of live ovules from ‘Hanfu’ × ‘RallsJanet’, ‘Hanfu’ × ‘Hanfu’, and ‘Hanfu’ (non-pollination). Then, embryos were visibly growing at 8 DAP, and the shape of globular embryos became clearer at 20 DAP. By 32 DAP, visible heart-shaped embryos with distinct outlines had formed in ‘Hanfu’ × ‘RallsJanet’ (alive) and ‘Hanfu’ × ‘Hanfu’ (alive) ovules. Nevertheless, in ovules of ‘Hanfu’ × ’RallsJanet’ (aborted), ‘Hanfu’ × ‘Hanfu’ (aborted), and ‘Hanfu’ (non-pollination), unfertilized egg cells were still apparent until 16 DAP. After 16 DAP, the ovule gradually dries out and it is difficult to observe the complete embryo sac through the DIC (Figure 1).

**Figure 2:**
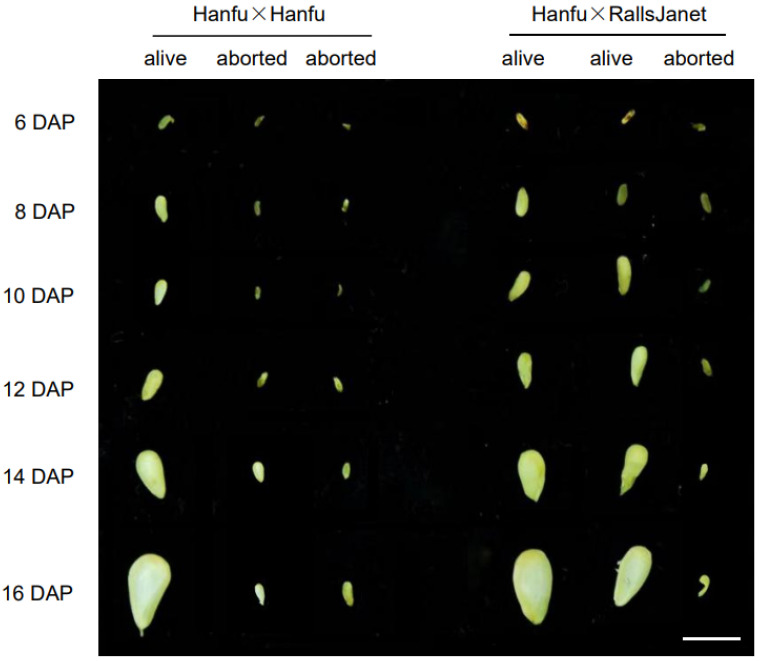
Observations on seed size of ‘Hanfu’ × ‘Hanfu’ and ‘Hanfu’ × ‘RallsJanet’ pollination combinations. Scale bars, 1 cm.

The ovules at 12 DAP were used to assess whether they were abortive or not. A total of 500 ovules were used from each self- and cross-pollinated ‘Hanfu’ combination. The results showed that in the ‘Hanfu’ × ‘Hanfu’ combination, live embryos could be observed in 171 ovules, accounting for 34.2% of the total, while the egg cells of 329 ovules, accounting for 65.8%, failed to be fertilized. In ‘Hanfu’ × ‘RallsJanet’, live embryos could be observed in 367 ovules, accounting for 73.4%, while 133 ovules had fertilization problems, accounting for 26.6%. These results suggest that the underlying cause of ovule abortion is failed embryo formation in ‘Hanfu’. In order to further explore the period of ovule abortion, developing seeds were removed from the ovaries at different times. There was no significant change in the appearance and size of seeds from the two pollination combinations before 6 DAP. At 8 DAP, a more obvious increase in the seed size of live ovules was observed; however, there was no significant change in the seed size of aborted ovules until 16 DAP (Figure 2). Seed size at 8 DAP was used to assess whether the ovules were abortive or not. After counting the seeds in each of the self- and cross-pollinated ‘Hanfu’ combinations, we found that there were 159 live ovules in the ‘Hanfu’ × ‘Hanfu’ combination, accounting for 31.8% of the total, while 341 ovules were abortive, accounting for 68.2%. There were 336 live ovules in the ‘Hanfu’ × ‘RallsJanet’ combination, accounting for 67.2% of the total, and 164 ovules were in the abortive state, accounting for 32.8%. The maximum cross-sectional area of ovules was also measured at each period; it was found that in live ovules, it gradually increased, starting from 8 DAP, while the size of abortive ovules did not change significantly until 16 DAP when they started to dry out. We can thus conclude that apple ovules are aborted before 8 DAP due to failure of embryo formation.

### 3.2. Sperm–Egg Cell Fusion Failure Results in Failure of Embryo Formation

**Figure 3:**
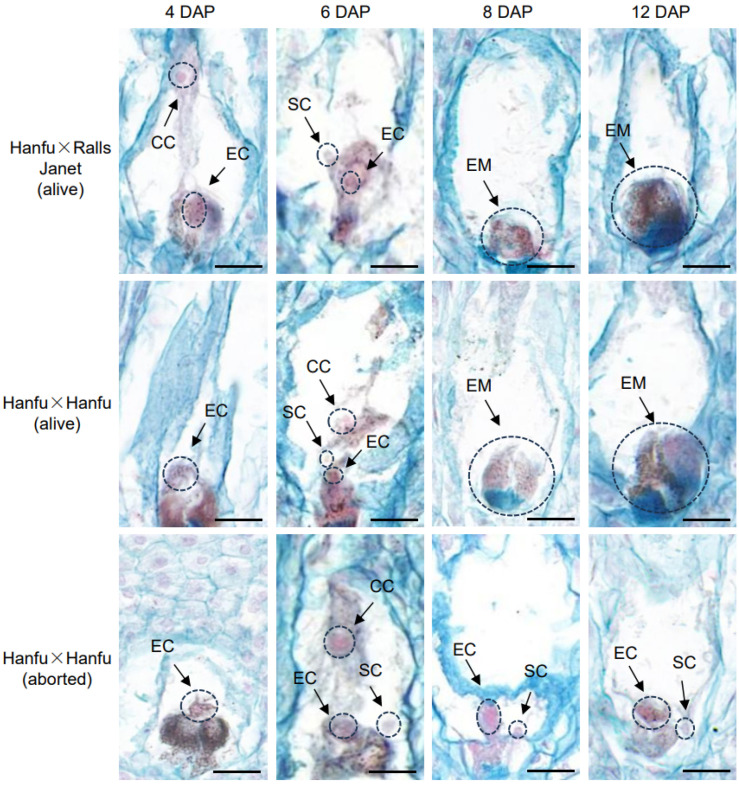
The process of sperm–egg fusion after pollination in ‘Hanfu’ × ‘RallsJanet’ (alive), ‘Hanfu’ × ‘Hanfu’ (alive), and ‘Hanfu’ × ‘Hanfu’ (aborted). DAP, days after pollination. SC, sperm cell; CC, central cell; EM, embryo. Scale bar, 100 μm.

To explore the reasons for embryo formation failure, paraffin sections of ovules from ‘Hanfu’ × ‘Hanfu’ and ‘Hanfu’ × ‘RallsJanet’ were observed at different times after pollination. At 4 DAP, sperm cells had not yet entered the embryo sac; both ‘Hanfu’ × ‘Hanfu’ and ‘Hanfu’ × ‘RallsJanet’ ovules displayed clear structures of unfertilized egg cells and central cells. At 6 DAP, sperm cells could be observed in the sac, most of which had not yet fused with the egg cells. Sperm cells in ‘Hanfu’ × ‘Hanfu’ (abortion) were located in the apical position of the synergid cell, while other sperm cells were located near the egg cell. At 8 DAP and 12 DAP, ‘Hanfu’ × ‘Hanfu’ (alive) and ‘Hanfu’ × ‘RallsJanet’ (alive) ovules had completed fertilization, their synergid cells had degenerated, and embryos could be observed. However, in ‘Hanfu ’× ‘Hanfu’ (abortion) ovules, sperm cells were still at the apical position of the synergid cell and had not moved toward the egg cell (Figure 3). Using the position of the sperm cell relative to the egg cell to assess abortion, the proportion of aborted ovules to live ‘Hanfu’ ovules at 6 DAP was 63% and 37%, respectively. At 8 DAP, this proportion was 66% and 34%. This suggests that ovule abortion in ‘Hanfu’ apples is caused by embryo formation failure, and its underlying cause is that sperm and egg cells are not fused normally at the early stage of fertilization.

### 3.3. Screening of Genes Related to Ovule Abortion in Apple

**Figure 4:**
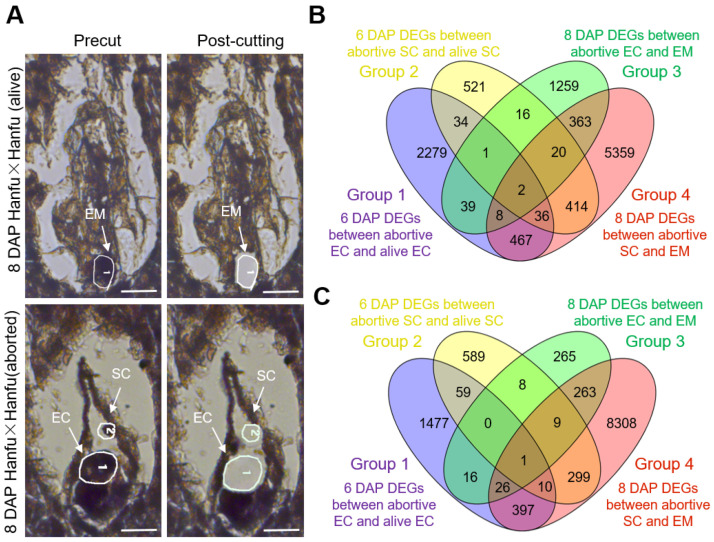
(**A**) Alive and abortive ovule at 8 DAP. On the left, sections before LAM cutting; the right shows the sections after LAM cutting. Scale bars, 100 μm. (**B**) Venn diagram of co-up-regulated expressed genes. (**C**) Venn diagram of co-down-regulated expressed genes.

To further explore the genes regulating the sperm–egg fusion process in the ‘Hanfu’ cultivar, egg cells, sperm cells, and embryos were excised separately from ‘Hanfu’ × ‘Hanfu’ (alive) and ‘Hanfu’ × ‘Hanfu’ (aborted) ovules using LAM. Subsequently, SMART-seq was performed. We obtained sperm and egg cells from ‘Hanfu’ × ‘Hanfu’ (aborted) ovules at both 6 and 8 DAP, whereas sperm and egg cells and embryos were obtained from ‘Hanfu’ × ‘Hanfu’ (alive) ovules (Figure 4A).

Genes with log2FoldChange > 2 were recognized as differentially expressed genes (DEGs). DEGs resulting from the comparison of abortive and alive egg cells at 6 DAP were named Group 1; DEGs resulting from the comparison of abortive and alive sperm cells at 6 DAP were named Group 2. Similarly, the DEG combinations obtained in the comparison of abortive egg cells and embryos at 8 DAP were termed Group 3, and those obtained in the comparison of abortive sperm cells and embryos at 8 DAP were termed Group 4.

In Group 1, the number of up-regulated genes was 2866; in Group 3, it was 1708; and 50 genes were up-expressed in both Group 1 and Group 3. A total of 1044 genes were up-regulated in Group 2; 6669 in Group 4; and 462 genes were up-expressed in both Group 2 and Group 4 (Figure 4B). In Group 1, the number of down-regulated genes was 1986; in Group 3, it was 588; and 43 genes were down-regulated in both Group 1 and Group 3. As for Group 2 and Group 4, there were 975 down-regulated genes in the former and 9313 in the latter, while 319 genes were down-regulated in both Group 2 and Group 4 (Figure 4C). The sequencing results showed that DEGs were more concentrated in egg cells at 6 DAP and in sperm cells at 8 DAP.

### 3.4. Functional Analysis and Validation of Genes Related to Ovule Abortion in Apple

**Figure 5:**
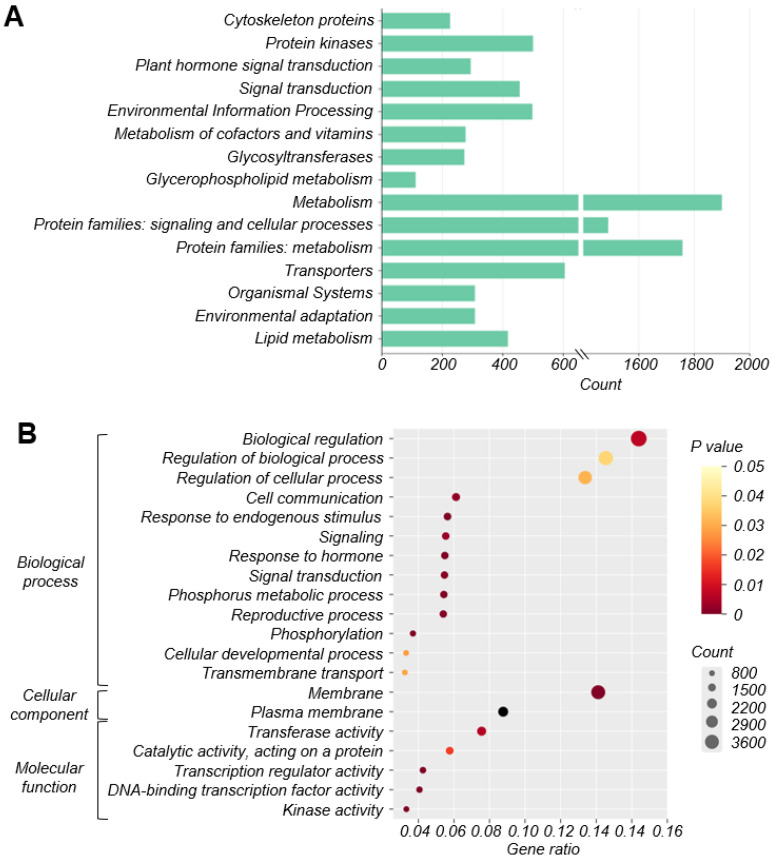
(**A**) KEGG enrichment analysis. Numbers in the horizontal coordinates represent the numbers of genes enriched in each pathway. (**B**) GO enrichment analysis.

To clarify the biological functions of differentially expressed genes during ovule abortion within the four groups, KEGG analysis and GO annotation were performed. The top 15 terms used for enriching those detected DEGs were generated and then clustered into metabolism, protein families: signaling and cellular processes, and protein families: metabolism. In addition, they were significantly enriched in pathways related to protein kinases, signal transduction, environmental information processing, transporters, plant hormone signal transduction, and lipid metabolism (Figure 5A).

According to the GO annotation, in the biological process (BP), the main DEGs were associated with biological regulation, regulation of biological process, regulation of cellular process, cell communication, and signaling. The DEGs distributed in the cellular component (CC) focused on membrane and plasma membrane, whereas those in the molecular function (MF) were mainly associated with transferase activity, catalytic activity, transcription regulator activity, DNA-binding transcription factor activity, and kinase activity (Figure 5B).

**Figure 6:**
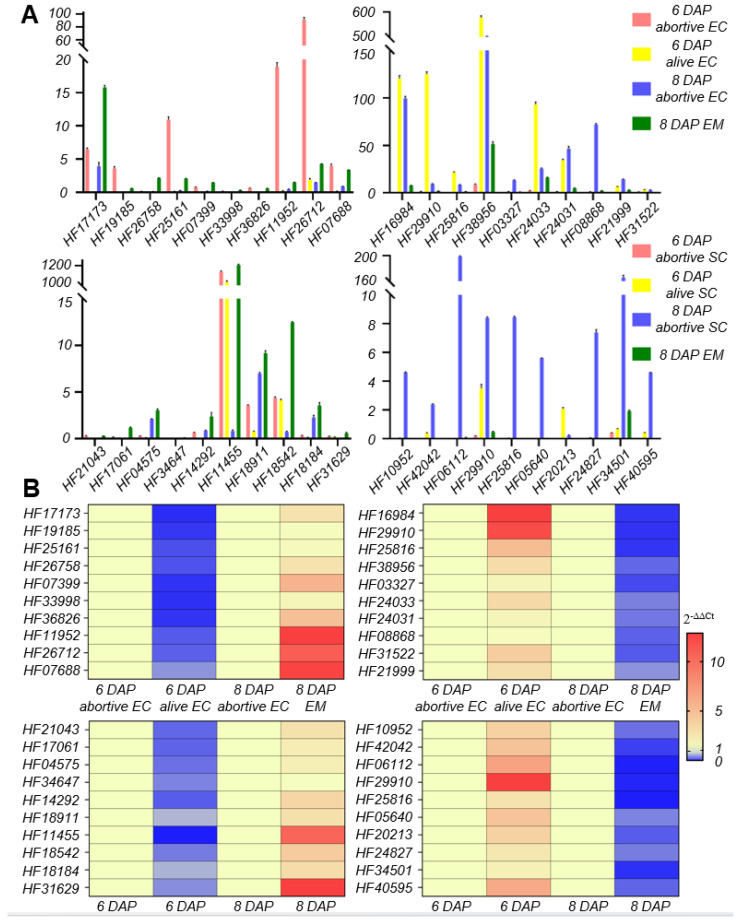
(**A**) FPKM histograms of the top 10 co-up-regulated DEGs in Group 1 and Group 3, co-down-regulated genes in Group 1 and Group 3, co-up-regulated genes in Group 2 and Group 4, and co-down-regulated genes in Group 2 and Group 4. (**B**) Heatmaps for validation of the top 40 DEG expression patterns. DAP, days after pollination. SC, sperm cell; EC, egg cell; EM, embryo. The results show the mean *±* SD and are based on three technical replicates. Data were analyzed using unpaired two-tailed Student’s *t*-test.

The aforementioned suggested that the process of fertilization involves many protein kinases. The top 10 genes in log2FoldChange were taken from each of the following four gene combinations: up-regulated in both Group 1 and Group 3; down-regulated in both Group 1 and Group 3; up-regulated in both Group 2 and Group 4; and down-regulated in both Group 2 and Group 4. The FPKM (fragments per kilobase of transcript per million mapped reads) of these 40 genes were counted (Figure 6A) and verified using qPCR (Figure 6B). It was thus determined that the 40 genes had the same trend of expression change during sperm–egg fusion as that found in the RNA-seq data, and were therefore deemed credible(Full-length primers and qPCR primers of the top40 DEGs can be found in the Supplementary Material).

## 4. Discussion

Apples exhibit S-RNase-mediated self-incompatibility and typically require cross-pollination in nature. However, there are a few varieties, such as ‘Hanfu’, which produce fruit after self-pollination, greatly reducing management costs, due to which they are widely grown in northern China. Despite these advantages, the self-pollinated fruits of ‘Hanfu’ apples exhibit a high rate of ovule abortion, which causes deformities in fruit. Therefore, investigating the mechanism of self-pollinated ovule abortion in ‘Hanfu’ apples and addressing the issue of malformed fruits are significant for improving fruit quality.

In this study, the observation of seed size confirmed the occurrence of ovule abortion in ‘Hanfu’ apples. Differences in size between alive and abortive ovules were observed from 6 DAP onwards, and the size disparity increased over time. Subsequently, a DIC microscopic observation system was established for observing apple ovules. It was found that the failure of embryo formation was the cause of ovule abortion in ‘Hanfu’ apples. With the presence or absence of abortive embryos as the assessment criterion, 500 ovules were examined. The proportion of abortive seeds among them was 65.8%, which was consistent with the observed ovule abortive rate of 68.2%. To investigate the reasons for embryo formation failure, paraffin sections of the ovules were observed. It was discovered that from 6 DAP to 12 DAP, egg cells within abortive embryos remained unfused, while those in alive embryos fused normally at 8 DAP and developed into embryos (Figure 3). Previous studies have shown that pollen tubes in *Arabidopsis thaliana* and *Torenia fournieri* enter the embryo sac through the synergid cell, and eject sperms to the chalazal edge of the degenerated synergid cell aided by the dynamics of cytoplasmic flowing [43]. After a brief pause, the sperm cells move toward the egg cell and central cell. Ovule abortion in ‘1913’ pears [27] was due to unfused sperm and egg cells, while in grapes, the cause of ovule abortion was programmed cell death [39]. This study suggested that the sperm cell movement pattern in apple is the same as that in *Arabidopsis thaliana*. In ‘Hanfu’ apple, ovule abortion was caused by a failure of embryo formation, with the fundamental reason being the failure of fusion between the sperm and egg cell—which is the same as that in ‘1913’ pears—occurring between 6 DAP and 8 DAP. As we know, cross-pollination may fail to fertilize the ovule. But self-pollination may still attach to the stigma if emasculation was not performed before cross-pollination. The documented cases of embryo formation failure in cross-pollination may then be partly due to self-pollination. We emasculated and bagged the inflorescences used in all three pollination combinations roughly one week before anthesis. At this moment, the anthers are still a long time from drying and almost all of the pollen has not yet been released. We hope to reduce the impact of self-pollination by doing this. DEG function was screened using KEGG and GO enrichment analysis. The results suggested that numerous protein kinases are involved in sperm–egg fusion; these genes may be located in the upstream pathway of the four genes that have been reported (Figure 5A,B). Of the 40 genes with the greatest differences in expression, HF19185 is a homoserine kinase, HF25161 and HF07399 are zinc finger proteins, and HF36826, HF11952, HF17061, HF14292, HF18184, and HF31629 are uncharacterized proteins. The genes whose expression was up-regulated within all four combinations were HF03602 and HF39852 (Figure 4B). HF03602 is a protein disulfide isomerase (PDI) with three structural domains, which are ERGIC_N, Thioredoxin and COPIIcoated_ERV. Previous studies have shown that PDI is mainly responsible for catalyzing the formation and cleavage of protein disulfide bonds and plays an important role in protein folding, modification, and secretion, among others. In *Arabidopsis thaliana*, PDI7 localizes to the endoplasmic reticulum (ER) and Golgi membranes in wild-type root tip cells, and was also detected in vesicles. In maize, the major PDI accumulates to high levels in seeds producing mutant storage proteins that trigger induction of the ER stress response [44,45,46,47]. HF39852 in *Arabidopsis thaliana* is a member of the oligopeptide transporter (OPT) family, but not of the ABC or PTR membrane transporter families. It is mainly responsible for the transportation of oligopeptides containing three to six amino acid residues. In previous studies, AtOPTs were preferentially expressed during seed germination, vegetative growth, and reproduction. For example, AtOPT1, AtOPT3, and AtOPT8 were uniquely expressed in pollen, while only AtOPT6 was expressed in ovules [48,49,50].The gene that was down-regulated and expressed within all four combinations was HF29910 (Figure 4C). As a heat stress protein in plants, this gene is mainly involved in the stress response to temperature [51]. However, whether and how these genes play a role in the process of sperm–egg fusion needs to be further investigated in the future.

## 5. Conclusions

In this study, we investigated the ovule development process and the mechanism of ovule abortion after self-pollination in apples. We found that abortion occurs before embryo formation as a consequence of fertilization failure. We identified the cause of ovule abortion as the lack of fusion between sperm and egg cells resulting in the embryo failing to form. Using paraffin sectioning and microscopic observation, it was found that 8 DAP was the best period for studying ovule abortion in ‘Hanfu’. Furthermore, using LAM and SMART-seq, we found and screened a number of genes that might be involved in sperm–egg cell fusion. Most genes related to ovule abortion are enriched in pathways related to protein kinases, signal transduction, environmental information processing, transporters, plant hormone signal transduction, and lipid metabolism. We can thus preliminarily clarify the mechanism of ovule abortion in self-pollinated apple fruits and provide a gene reserve that will aid in further study and help improve apple fruit quality.

## Data Availability

The data presented in this study are available upon request from the corresponding author.

## Supplementary Materials

The following supporting information can be downloaded at: https://www.mdpi.com/article/10.3390/plants13070996/s1, Table S1. Full-length primers of the top40 DEGs; Table S2. qPCR primers of the top40 DEGs.

## Abbreviations

DAP, days after pollination; LAM, laser-assisted microdissection; DIC, differential interference contrast; SMART-seq, switching mechanism at 5′ end of the RNA transcript; SC, sperm cell; CC, central cell; EC, egg cell; EM, embryo; DEGs, differentially expressed genes; FPKM, fragments per kilobase of transcript per million mapped reads.
