# Supplementary material for: The Mechanism of Ovule Abortion in Self-Pollinated ‘Hanfu’ Apple Fruits and Related Gene Screening
Source: Plants (Basel). 2024 Mar 30;13(7):996. doi: 10.3390/plants13070996 (PMC11013273; doi:10.3390/plants13070996)
Supplement: Supplementary file 1 [file plants-13-00996-s001.zip › plants-2879689-supplementary.pdf]

Table S1

Full-length primer of the top40 DEGs

	F	R
HF17173	ATGGAGGCTTGTGTGGTG	CTAAGGGAAAGAAGCCAAAGC
HF19185	ATGGCAATTTGCCACCAA	TTAGTTCAATAATATTCCTATTATTTTG
HF26758	ATGGTTTATTCTGCCCAGCAC	TTAAAAGTAGGTACTAACTGATTCC
HF25161	ATGAAGAGAAGCATCGCAGATG	TTAGAAAAACCCAACAAGTGGAGC
HF07399	ATGCAATCGGAAACCAATACCG	CTAAGCCTCACTCCTAGAAGAT
HF33998	ATGGTAGTTATCGAAGATATAGCAGC	TCATTCCTCCTTTTTGTCAAGG
HF36826	ATGGGCAACTACATTTCTTGTAGC	TCACCTTGAAGAAACAGAAACTGC
HF11952	ATGTCTGATTCTAATTCTACACCTGC	TTAGTAAGGCAAGCTCTTAGAGC
HF26712	ATGGCTGAGCGCAATAACTCA	TTACGCTGCATCATTTGACCC
HF07688	ATGGCTTCGACTTCCTCTTTG	TTAATTTCTAGGTGTAGAGTCCACC
HF21043	ATGAAGACAATTAGTCTCATTGATCTTTC	TCAGTAAATTTTGACAAAGATGCCCTC
HF17061	ATGTCTCTTAGACCAAGAGCAAC	TTATTTGCCAGGCGTAAGAGG
HF04575	ATGGAGGGTTTGATTCCTTTTGTG	TTAGTTACTAGCAACTCGGTG
HF34647	ATGGATGGGATGGACTTAACAC	TCAGCCCTTTCTAATCTTTGACTTG
HF14292	ATGGAGAGGCCGGAAGGAT	TCATTTGGCCGGTTTACTACC
HF11455	ATGAAGATGGAGCAAAATTCAACCCTC	CTAGGTGCAGGTGGAGGGA
HF18911	ATGGGTGTTGGAATTCAAACAATGTG	TCAGACTCTTACTCTTTCGACT
HF18542	ATGGCAAGGAACACAAACATGG	TTAATTAAAGCTATAGAAGAAGATGGCC
HF18184	ATGGCTGACTACGAGTTTC	CTAAGCTTCCAAGAAACCAGTC
HF31629	ATGGCAATGGATAAGGATAACATACTG	CTAGTTCTCTTTCATCGCGGAT
HF16984	ATGCATATGAAGAATAACGTGATGATGG	TCATGCGACCTTTGTAGGTTC
HF29910	ATGAGGAAACAACCTTCTCGAAATATTC	TTACAGCTCTTGCTTGGACTG
HF25816	ATGTCTTCTGTCGATGTTCCAC	TTATAATGCCACAGGTTCCAC
HF38956	ATGTCACTCATTTCCCAAGTG	TTAATTTCCTTCAATCTGAATAACCTTC
HF03327	ATGGAGCAAGAGATGGTGATG	CTAAGGGTACTTCTTCTTTGTAACAC
HF24033	ATGGCTCTCAGTCTCTTTGTC	TCACACAAACAGAAAGGCACAG
HF24031	ATGGCTCTCAGTCTCTTTGGC	TTAGCCGGAGATGTCAATGG
HF08868	ATGGCCCTTCAACACACTTTG	TCAAACTTTGGTGGAGGCATC
HF21999	ATGGGATCCCGAACAAACGT	CTAGGCATTATCATTCAGGAATTTT
HF31522	ATGGGCAGAGCTCCTTGC	TTATTTGAGGCCAAAGTAGGTTGC
HF10952	ATGGATCGACCAGGAACG	TTAAAGCTCTTGCTTGGGCTC
HF42042	ATGTTTACTTATTTGGCTGCTTGG	TTAAGAATATTTGTAATCAGTCTGACGAT
HF06112	ATGGCTCCTCTCATCTTC	TCAGCATTCGGGTGTTAGTG
HF05640	ATGGCCTCAACCTCCTCC	CTAGCTGCTGCCGATTATATC
HF20213	ATGCAGACGACTACTACAACC	TTATTTTCCTGCAGACTCCTTTGG
HF24827	ATGGCAATTCTTGGTTTTCTCTTGG	TTAGAATTGTTGGCCGCTGAATGT
HF34501	ATGCATGTGAAGAAGAATAATGC	TCATGCGACCTTTGTAGG
HF40595	ATGATTTCTTCAAGCATTTCTCAG	TCAAAGTGTAACGAAATTTTTAACA

Table S2

qPCR primer of the top40 DEGs

	F	R
HF17173	TGGCTCATCTCCCTTACCCT	AAAGCCACCTCCAAAAGGCA
HF19185	TCTCGACCCATCCTCACCAT	TGAAATGGAGATCTCGCCCG
HF26758	CCACCTCACTACCACACCAC	AACTCGATGGCCGGAATGTT
HF25161	CCCAGGTTAACCGGACCATC	CGCCTGCCCTATAGCAAACT
HF07399	CAAGCACTAGGTGGGCATCA	GCTCGAGGGCCCATAACTTT
HF33998	TGACTGGAGGGTTTGTGTCG	CCTTCTCGATCTCATCCGCC
HF36826	CTGATGGTCGACACCCCAAA	AACTCTTCGTCGGCACTGAG
HF11952	AGCTCACCAAAGCTCAGACC	GGTGAACCGATTGGAACCCT
HF26712	TTCGCGCGAGGCATATAAGT	TAGTTGTGGTTCGCGAGCTT
HF07688	CTGGTTGTGAGTCTGGACGG	TGCCTCTTGGAAGGACCAGA
HF21043	TTTCTCTGTTCTGACGGCCC	CCAGACGTCGGATAAGACCG
HF17061	AGCAAACCTAGCCAACCTCC	CGAAGCATCCGGGTAGTCAA
HF04575	GACCGCTTCGAATCATCGGA	GCAACTCGGTGACGAAATGG
HF34647	GCACCTGATTCCACAGTCCA	TCCCAGTGCTGACGTCTTTC
HF14292	CCAAAGTTGTTGGCGTTGGT	CGCTCAGACTCCGTTGCATA
HF11455	GTTCGGGTTCGGATTACGGT	ACCTTCCTTGGTGCTTCTCG
HF18911	GCTGCTGCTTTCCTGCATTT	GCCACATCTCAGGAGTGCTT
HF18542	GCAGTGCCGGTCAGAAATTG	AACGGTCCAAGTGACAGGTG
HF18184	GGCGTCACCATCAAAACGAC	GACCGGCACCGACTTGATAA
HF31629	CTCGGCCTACAAACCCTCTC	TCCTTCGCGTCTAATGCTCC
HF16984	AGGGCGACAACTACAAGTGG	TCTGATCCAAGTCGGCGTTC
HF29910	TCGCCTTGAGCTTTCTGCTT	GAGTGCCAGCAGAGTGTCTT
HF25816	GCAGTGAAGGCTGCATCAAC	CGCTTCTTCTTTTGTGGCCC
HF38956	CCCTAGACGCCCATGTGTTT	CTCCTTAGGAACTTGCCCCG
HF03327	AGCCATTCGACCAGTCACAG	TCTACCTCCGAGTGCGGTTA
HF24033	CTCTGCAGACCGTTCCGTT	TGGCACAGAAGGTTTCTCCG
HF24031	GCTGAGCTCGTGTCTCCTAC	GTGGGTACCGGAGTGACTTG
HF08868	TGCAGTCGCATGGTTCTTCT	CTGCAGGGCTCATTTCTGGA
HF21999	CGCAGAGAGGATTCAGGACC	TGGCATCGACTCCAAGGTTC
HF31522	CATGGGCTGGACGATCAGAA	TACTGGTCCCTGCACTGCTA
HF10952	ATCACTGGCACCGAGTTGAG	CTTGGCAGGTTCACCCTGAT
HF42042	GGCGTGTTGCAGAGATTGTC	GTTTGGTTCGAAAGCCGCAA
HF06112	GGGCCACAGAGGATGGAAAA	CTCACTAAGCCCCGATGAGC
HF05640	CTCTAGTTGACGCCAAGGCT	TTCCCTGCACGGTTTTGAGA
HF20213	TGCAACGGCGTTTTATGCAA	CCTTGGGATCCCCACCTAGA
HF24827	TAGGTGTGTGAGTGACCCCA	ATCTGTTGGAAGACGGGCTG
HF34501	AGGGCGACAACTACAAGTGG	CGCAAACTTTGGCACCGTAA
HF40595	GAACTTCCGGGGAACCATGA	ATGGGCACATCATCCACCAG
